# Using BiVO_4_/CuO-Based Photoelectrocatalyzer for 4-Nitrophenol Degradation

**DOI:** 10.3390/ma13061322

**Published:** 2020-03-14

**Authors:** Thiago Martimiano do Prado, Fernando Lindo Silva, Guilherme Grosseli, Pedro Sergio Fadini, Orlando Fatibello-Filho, Fernando Cruz de Moraes

**Affiliations:** Department of Chemistry, Federal University of São Carlos, São Carlos-SP 13565-905, Brazil; thipra@gmail.com (T.M.d.P.); fernandolindosilva@gmail.com (F.L.S.); guilhermegrosseli@gmail.com (G.G.); psfadini@gmail.com (P.S.F.); bello@ufscar.br (O.F.-F.)

**Keywords:** bismuth vanadate, cooper oxide, 4-nitrophenol, photoelectrocatalysis, degradation

## Abstract

The present work reports the degradation of 4-nitrophenol using BiVO_4_/CuO hybrid material synthesized by the precipitation of BiVO_4_ in the presence of CuO. Morphological and structural characterizations were performed using X-ray diffraction and scanning electronic microscopy coupled to energy dispersive X-ray spectroscopy. Through the calculation of the Kubelka–Munk function applied to diffuse reflectance spectrophotometry data, the hybrid material presented absorption edge of 1.85 eV. The formation of *p-n* heterojunction between BiVO_4_ and CuO renders the hybrid material suitable for the construction of a photoanode employed in hydroxyl radical generation. UV–vis spectrophotometry and high-performance liquid chromatography experiments were performed in order to monitor the degradation of 4-nitrophenol and the formation of secondary products. Additional information regarding the hybrid material was obtained through ion chromatography and total organic carbon analyses. The application of BiVO_4_/CuO-based photocatalyzer led to a 50.2% decrease in total organic carbon after the degradation of 4-nitrophenol. Based on the results obtained in the study, BiVO_4_/CuO has proved to be a promising material suitable for the removal of recalcitrant compounds in water treatment plants.

## 1. Introduction

The growing rise of the global population and changes in consumption patterns have led to a dramatic increase in the presence of chemical contaminants in food and water; this has resulted in increasing demands for the development of new technologies capable of degrading these contaminants and helping to ensure the safety and security of food and drinking water consumed by the global population. In this context, socio-economic activities have increasingly been geared towards the promotion of productivity efficiency in farming, water catchment, and treatment [1,2]. Activities conducted in agricultural industry can be divided into two different sectors: agricultural and livestock sectors. The first sector has high economic importance in terms of sustainability and trade balance between consumer and producer countries [3,4,5]. To attend the internal demand and exportation needs, producer countries invest in measures that enhance productivity by reducing losses associated with diseases and pests that affect crop production [6]. In this case, the use of pesticides is directly related to these preventive measures. Despite the positive effects of disease and pest control, the use of pesticides causes environmental degradation, including the contamination of soil and water bodies [7].

The presence of pesticides among environmental contaminants has been addressed in studies related to the impacts of these contaminants on human health. Pesticides are synthetic substances—used in agriculture as insecticides, herbicides, fungicides, and molluscicides—whose residues have been found in drinking water, food, and water bodies [8,9,10]. These substances belong to the largest group of substances classified as endocrine disruptors [8,9,10]. Endocrine interferers belong to a category of environmental contaminants that cause changes in the behavior and functioning of the endocrine system. The concentration of these substances in the environment varies from µg L^−1^ to ng L^−1^ [11]. 4-nitrophenol (4-NP) is a hazardous substance regarded as a sub-product of hydrolysis reaction of organophosphate pesticides (e.g., parathion and metyl-parathion), and which has been reported in studies related to risk assessment of environmental contamination [12,13,14,15]. This compound is used as precursor of other molecules, including other types of organophosphate pesticides (such as fenitrothion), drugs, dyes [16], and artificial sweeteners (e.g. dulcin) [17]. Most of these substances are persistent in the environment, and when present in water sources the molecules are not eliminated in water treatment plants (WTP). 

Conventionally, sewage treatment plants (STP) are constituted by two types of treatment techniques [18]: physical and biological treatment techniques. The physical treatment technique is used for the removal of suspended solids; and the biological (aerobic or anaerobic) technique is concerned with finding the adequacy of the amount of organic matter. Different combinations of aerobic/anaerobic/anoxic processes can be useful for denitrification and phosphorus removal. Some studies have demonstrated the inefficiency of the biological technique for the treatment of sewage containing 4-NP which has persisted in the treated effluent [19,20]. Considering the need for enhancing the efficiency of STPs, the present work proposes an alternative process for 4-NP degradation. The mechanism proposed here involves the use of advanced oxidative process (AOPs) through the exploration of the properties of a semiconductor material for the photoelectrocatalysis of 4-NP. The expected reactions are represented by the Equations (1)–(5) below:

BiVO_4_/CuO + *hν* → *e*_cb_^−^ + h_vb_^+^ (1)

h_vb_^+^ + H_2_O → •OH + H^+^ (2)

H_2_O → •OH + H^+^ + e^−^ (3)

*Pollutant* + •OH → *Pollutant* • + H_2_O (4)

*Pollutant* • + O_2_ → *Chemically oxidized product* (5)

**Scheme 1:**
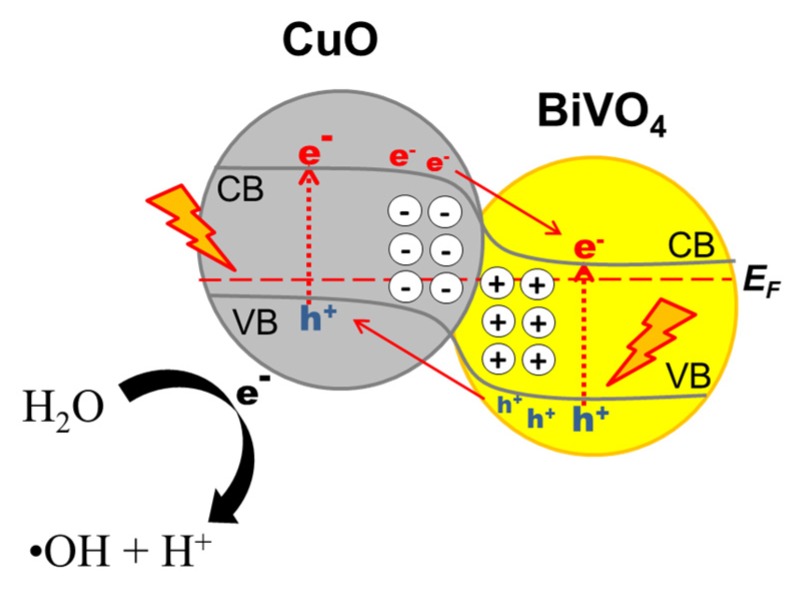
Mechanism for photoelectrocatalytic activity of BiVO_4_/CuO in •OH generation.

The BiVO_4_/CuO had heterostructure with p-n junction (Scheme 1), where CuO is a n-type semiconductor and BiVO_4_ is p-type. In this case, before the irradiation of light, the n-type semiconductor electrons tends to diffuse through the p–n interface for the p-type semiconductor, leaving a kind of positive charge. Meanwhile, the holes (h^+^) of the semiconductor p-type tend to spread to the n-type semiconductor, leaving a negatively charged species. The diffusion of e^-^ and h^+^ will continue until that the system’s Fermi level balance is achieved. Consequently, an internal electric field is generated in the region close to the p–n interface. The e^-^ and h^+^ photogenerated in p-type semiconductors and n will migrate under the influence of the internal electric field to the BC of the n-type semiconductor and to the BV of the p-type semiconductor, respectively, which results in spatial separation and increased life of the loads. The photogenerated holes in the valence band (VB), h_vb_^+^, and electrons in the conduction band (CB), *e*_cb_^−^, are represented in Equation (1). The holes in CuO CB promote the direct oxidization of water molecules adsorbed on the semiconductor surface (Equation (2)), leading to the production of hydroxyl radicals and hydrogen cation. Simultaneously, when the photoanode works at controlled potential, which is sufficient to promote water splitting, this process leads to the formation of hydroxyl radicals, hydrogen cations, and electrons (Equation (3)). The hydroxyl radicals produced in the processes depicted in Equations (2) and (3) react with the molecule of the pollutant organic compound, transforming into pollutant radicals (Equation (4)). The radicals are transferred to the dissolved O_2_ in solution, forming a chemically oxidized product (Equation (5)). The hydroxyl radical generation in the BiVO_4_/CuO surface is represented in the Scheme 1.

The combination of photocatalytic and electrolytic processes is referred to as photoelectrocatalysis (PEC). The PEC technique is a promising electrochemical advanced oxidative process (EAOP) regarded suitable for the degradation of organic pollutants present in environmental samples [21,22]. Electrochemical advanced oxidative processes (EAOPs) are found to have superior advantages over advanced oxidative processes (AOPs) in the sense that, unlike the latter, the EAOPs do not require the use of reagent oxidant compounds—such as chlorine [23], ozone [24], or hydrogen peroxide [25]—which tend to increase the total volume of the substances under treatment. The use of PEC for the treatment of water contaminated with 4-nitrophenol was proposed in this present study considering the significant presence of this compound in the environment. Specifically, 4-nitrophenol is found present in the environment either as byproduct of the hydrolysis of organophosphate pesticides; or discharged in industrial effluents, where it is used as precursor for the production of different kinds of compounds, from pesticides to artificial sweeteners. Under the PEC approach, the hybrid semiconductor BiVO_4_/CuO acts as photoanode, taking into account the photocatalytic property of the semiconductor material and its ability to withstand the application of high potentials aiming at promoting water splitting.

## 2. Experimental Methods

### 2.1. Materials

All reagents used in the experiments were of analytical grade; and they were employed without further purification. Ammonium vanadate (NH_4_VO_3_), 4-nitrophenol (4-NP), and sodium sulfate (Na_2_SO_4_) were purchased from Sigma-Aldrich. Bismuth (III) nitrate pentahydrate (Bi(NO_3_)_3_∙5H_2_O) was acquired from Chem-Impex, and sodium hydroxide (NaOH) was obtained from Acros Organics. Cooper (II) nitrate trihydrate (Cu(NO_3_)_3_∙3H_2_O) was acquired from Vetec. 0.1 mol∙L^−1^ NaOH was used as supporting electrolyte; and this electrolyte was also used for the preparation of the working standard solutions through the dissolution of 4-NP. The aqueous solutions were prepared with water purified in a Milli-Q System (Direct 8 - Millipore, Bedford, MA, USA), under resistivity ≥ 18 MΩ cm.

### 2.2. Apparatus

**Scheme 2:**
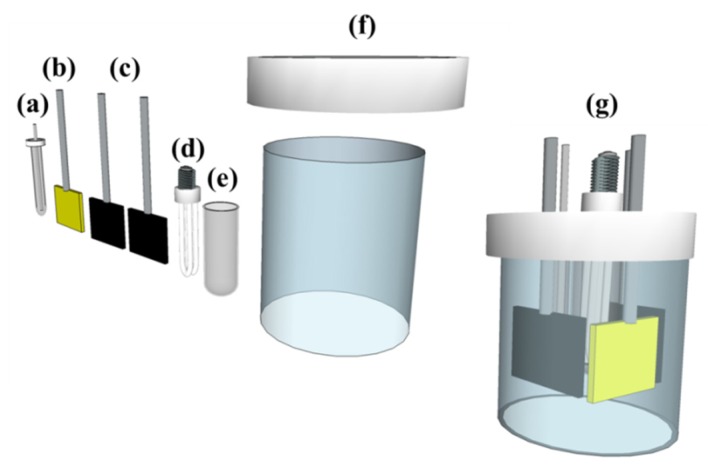
Representation of the electrolytic cell parts: (**a**) Ag/AgCl(KCl_sat_) reference electrode; (**b**) working electrode; (**c**) titanium platinized counter electrodes; (**d**) UV-C lamp; (**e**) quartz bulb; (**f**) reservoir; and (**g**) assembled electrolytic cell.

Electrolytic experiments were performed in a single compartment electrochemical cell with a 30 W UV-C lamp (Osram, Munich, Germany), as shown in Scheme 2. The system was kept under a controlled temperature at 20 °C using FTO/BiVO_4_/CuO as working electrode (geometric area = 20.25 cm^2^). Two platinized titanium foils (geometric area = 40.50 cm^2^, each) were employed as counter electrode and Ag/AgCl (KCl_sat_) was used as reference electrode. The assembled electrochemical cell was controlled by a potentiostat/galvanostat PGSTAT 302N Autolab (Ultrech, Ultrech, Netherland) operated using the software NOVA 1.11.1 and equipped with BOOSTER10A module Autolab (Ultrech, Ultrech, Netherland). 

The photoelectrochemical characterizations were carried out in an electrolytic cell with quartz window and assembled with photoanode, platinum foil counter electrode and Ag/AgCl (KCl_sat_) reference electrode. The photocurrent responses were obtained by linear sweep voltammetry (LSV) using potentiostat/galvonostat PGSTAT304 equipped with an Autolab module FRA32N (Ultrech, Ultrech, Netherland) and coupled to a light emission diode (LED) driver and white LED (44 W) Autolab (Ultrech, Ultrech, Netherland), as displayed in Figure S1 (Supplementary section). The rate of decolorization of the 4-nitrophenol solution was monitored at 400 nm using Varian Cary-50 UV–vis spectrometer. The quantification of 4-nitrophenol during the degradation process was carried out by high performance liquid chromatography (HPLC) using a chromatographic system, which consisted of a Shimadzu Prominence LC-20AT modular system with two CBM-20A pumps, a CTO-10AS oven, a SIL-20A auto sampler, a SPD-20A variable wavelength detector, and an LC-10 Workstation Class data processor (Shimadzu, Tokyo, Japan) with UV–vis detection, operated at 400 nm. The separation analysis was carried out using a Luna C18 column, 250 × 4.6 mm^2^ i.d., 5µm(Phenomenex, Torrance, CA, USA) and isocratic mobile phase composed of acetonitrile: water (50:50 v/v), where the water was acidified by acetic acid (99:1 v/v) at a flow rate of 1 mL∙min^−1^. The formation of ions during the degradation process was monitored by an ion chromatography system, which consisted of 881 Compact IC pro, 800 Dosino dosers and 919 IC Autosampler plus sampler (Metrohm, Herisau, Switzerland). The quantification of total organic carbon (TOC) relative to degradation was carried out using an organic carbon analyzer, model TOC-L (Shimadzu, Tokyo, Japan).

The morphology of the synthesized materials was investigated by scanning electron microscopy using microscope model JSM-6301F (JEOL, USA). X-ray diffraction (XRD) analyses were performed with the aid of a D8 Advance diffractometer (Bruker, USA), using CuK radiation l = 1.5406 Å, voltage of 40 kV, and theta-2theta configuration. Diffuse reflectance spectroscopy (DRS) experiments were performed in order to calculate the band gap energy (*Eg*) and absorption edge (*E_ae_*) values for the synthesized materials using UV–vis data obtained with the aid of Cary 5E spectrophotometer (Varian, USA).

### 2.3. Preparation of BiVO4/CuO 

CuO was prepared by thermal decomposition of Cu(NO_3_)_3_∙3H_2_O in an oven, under a period of 30 min at 300 °C, with heating rate of 20 °C min^-1^. A mass of 1.940 g of Bi(NO_3_)_3_∙5H_2_O was mixed with 0.600 g of CuO and suspended in 20.0 mL of ethyleneglycol by sonication for 40 min. Simultaneously, a mass of 0.470 g of NH_4_VO_3_ was dissolved in warm deionized water at 80 °C. The NH_4_VO_3_ solution was then dropped slowly, with constant stirring, into the bismuth/CuO suspension, forming a BiVO_4_/CuO suspension. Precisely 2.0 mL of BiVO_4_/CuO suspension was dropped on FTO surface (conditioned over a hotplate set to 180 °C). After a few minutes, a BiVO_4_/CuO plastic film was formed. This procedure was repeated three times until the FTO surface was completely covered. The FTO/BiVO_4_/CuO was annealed for 1 hour in an oven under a heating rate of 10 °C/min up to the temperature of 500 °C. This step was essentially required in order to transform the BiVO_4_ crystalline form from orthorhombic to monoclinic phase, since the semiconductor exhibits better optical activity in monoclinic phase [26]. Thereafter, the electrode was removed from the oven and allowed to cool to room temperature. 

## 3. Results and Discussion

### 3.1. Characterization of BiVO4/CuO

**Figure 1:**
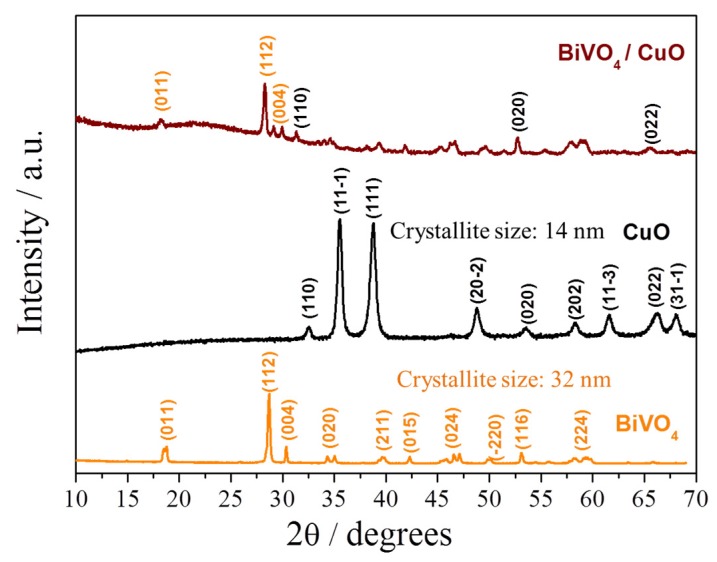
X-ray diffraction patterns of the BiVO_4_, CuO, and BiVO_4_/CuO.

The structural characterization of BiVO_4_/CuO was performed using X-ray diffraction. The XRD experiments were carried out for BiVO_4_ and CuO separately and for the hybrid BiVO_4_/CuO. The diffraction patterns obtained were compared with the information card from the Crystallography Open Database (COD) in order to confirm the formation of hybrid material with the features of each compound. Figure 1 shows the characteristic patterns of CuO and BiVO_4_, with both exhibiting monoclinic crystalline phase, based on the COD entries 1011148 and 909870, respectively. The formation of a hybrid material can be confirmed by the existence of the plane orientation in *hkl* directions 011, 112, 004 and 110, 020, 022, which are typically related to BiVO_4_ and CuO, respectively. The average crystallite sizes of the pure materials obtained by the Scherrer equation [27] were 14 nm and 32 nm for CuO and BiVO_4_, respectively. The lower intensity of the CuO diffraction peaks present in the hybrid material, when compared to the diffractogram obtained for the pure CuO, is due to this material being the constituent in lesser quantity in the hybrid material.

**Figure 2:**
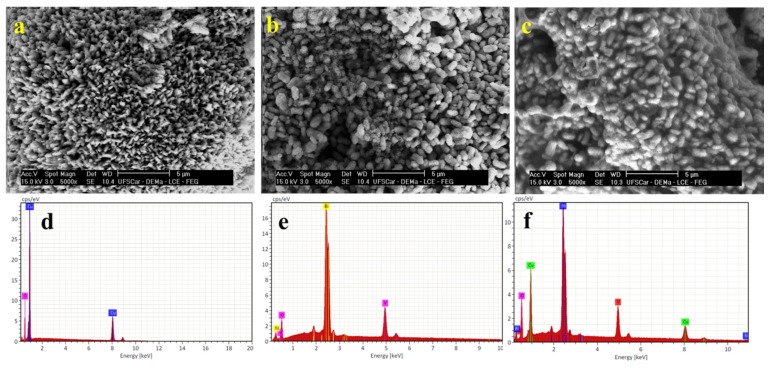
SEM micrographs of the (**a**) CuO; (**b**) BiVO_4_; and (**c**) BiVO_4_. Respective EDS data of (**d**) CuO; (**e**) BiVO_4_; and (**f**) BiVO_4_.

The morphological characterization of BiVO_4_/CuO was analyzed by SEM microscopy, as shown in Figure 2. Based on the micrograph presented in Figure 2a, one can observe the formation of polycrystalline grains of the monoclinic CuO, which is constituted by the agglomeration of nanocrystallites [28]. Figure 2b shows the typical worm-like morphology [26] of monoclinic BiVO_4_ formed by polycrystalline grains. The hybrid material shown in Figure 2c presents of BiVO_4_ and CuO grains, which is associated with the reduction of interstices compared to Figure 2a,b. Figure 2d–f show characteristic excitation peaks, corresponding to the elements present in each sample, obtained by the energy dispersive X-ray spectroscopy (EDS) technique. Based on the comparison of these data, one can confirm that the BiVO_4_/CuO has been synthesized successfully.

Optical characterizations were performed by DRS (diffuse reflectance spectroscopy); and the application of the Kubelka–Munk [29] function allowed the calculation of the estimated *E_g_* of the isolated materials and the *E_ae_* of the hybrid material, through the extrapolation of linear region from the Tauc plot, as shown in supplementary section (Figure S2). The *E_g_* values obtained for CuO and BiVO_4_ were, 1.36 eV and 2.03 eV, respectively; these values are approximately similar to those reported in the literature [30,31,32]. By contrast, the BiVO_4_/CuO presented *E_ae_* = 1.85 eV, which is within intermediate value of each pure material band gap. These results demonstrated the hybrid characteristic of BiVO_4_/CuO with an absorption edge suitable for its use as photoanode operating under visible or UV illumination.

### 3.2. Photoelectrocatalytic Performance of FTO/BiVO4/CuO

**Figure 3:**
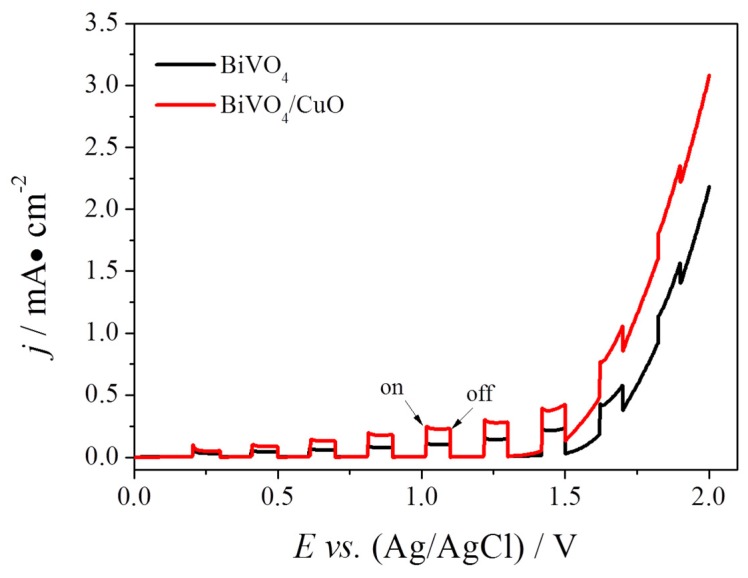
Linear sweep voltammograms of BiVO_4_ and BiVO_4_/CuO. Experiments performed in 0.1 mol∙L^−1^ Na_2_SO_4_ at scan rate of 50 mV∙s^−1^.

Linear scan voltammetry experiments were carried out with intermittent illumination (on/off operation) on the photoanode from a white LED light, λ > 410 nm and 44 W. Figure 3 presents the voltammograms for the photoanode modified with BiVO_4_ and BiVO_4_/CuO; here, one can observe the presence of photocurrent for both of the materials. By data comparison, one can conclude that the presence of CuO contributed to an increase in photocurrent signal in all the scanned potential range, including the water splitting region from the onset potential of +1.4 V to +2.0 V. This outcome can be attributed to the fact that CuO is a *p-*type semiconductor whose valence (VB) and conduction (CB) bands overlap those of BiVO_4_, which is an n-type semiconductor. This behavior leads to the formation of *p-n* heterojunction, favoring the charge transfer between VB and CB and a decrease in the electron–hole recombination rate of the hybrid material [33]. These results are in line with the data obtained in the DRS analysis; essentially, the results indicate that the presence of CuO in the hybrid material plays a vital role by increasing the photocatalytic activity of BiVO_4_. 

### 3.3. Photoelectrochemical Degradation of 4-Nitrophenol

**Figure 4:**
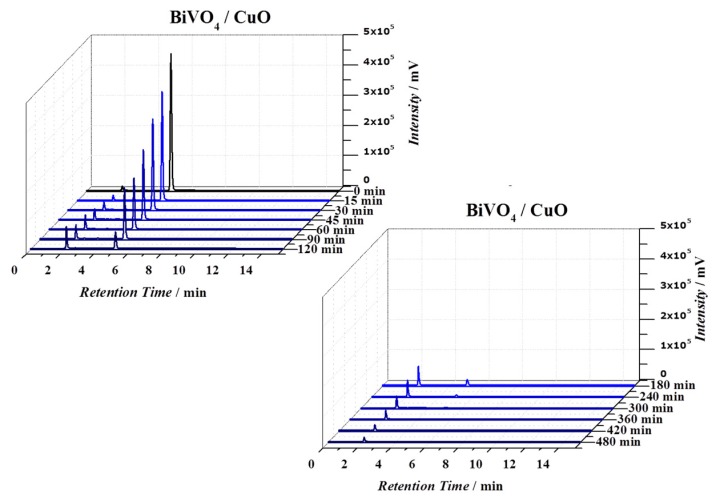
Chromatograms obtained by HPLC of samples collected during the 4-nitrophenol degradation experiments at different interval times.

To conduct the photoelectrodegradation experiments, a solution containing 30 mg∙L^−1^ of 4-nitrophenol was prepared with 0.1 mol∙L^−1^ NaOH. The use of alkaline electrolyte helps to avoid the leaching of BiVO_4_ by ensuring the stability of the compound. The photoanode was maintained under UV-C illumination at controlled potential of +4.0 V for 8 h. The photoelectrocatalytic process exhibited an average current density of 23.0 mA∙cm^−2^; the process was monitored by UV–vis spectrophotometry. After 4 h of analysis, the solution was found to have undergone discoloration and a decrease in concentration was observed in the absorbance versus time curve (see Figure S3). In order to accurately evaluate the degradation process, HPLC analysis was carried out aiming at monitoring the decrease of 4-nitrophenol over time. For the accurate determination of the analyte, a calibration curve was constructed using a stock solution of 100 mg∙L^−1^ of 4-NP, which was diluted to 0.50 mg∙L^−1^ 4-NP. The linear correlation obtained presented values of *R^2^* = 0.999, −0.48, and 1.48 × 10^−5^ for the intercept and slope, respectively. Figure 4 shows the chromatograms of the samples in different degradation times. As can be noted, an increase in high intensity is observed for the characteristic signal of 4-NP in the initial samples; however, over time, a decrease is observed in the characteristic signal of 4-NP concurrently with the formation of a secondary product. At the end of the degradation process, the peak corresponding to 4-NP is found to have practically disappeared, while a significant decline in intensity is observed for the signal related to the secondary product. 

**Figure 5:**
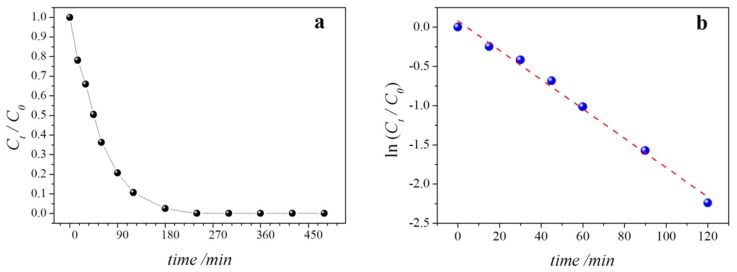
(**a**) Relative concentration decay of 4-nitrophenol determination by HPLC during degradation experiments. (**b**) Linear region used for the calculations of rate constant from pseudo-first order reaction.

The degradation kinetics was determined for the results in Figure 5a within the time range in which a rapid decline is observed in 4-NP degradation (i.e., 0–120 min). Here, a pseudo-first order mechanism was considered, and the rate constant *k* = 3.11×10^−4^ s^−1^ was calculated by the slope of linear regression of absolute value of ln(*C_t_/C_0_*) versus time as presented in Figure 5b. 

**Scheme 3:**
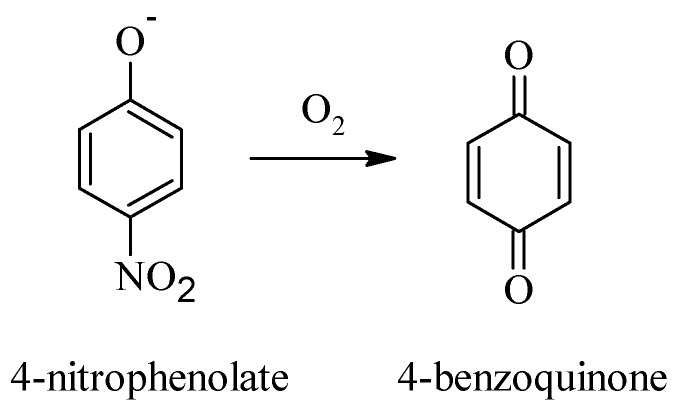
Mechanism for oxidation of 4-nitrophenolate.

**Scheme 4:**
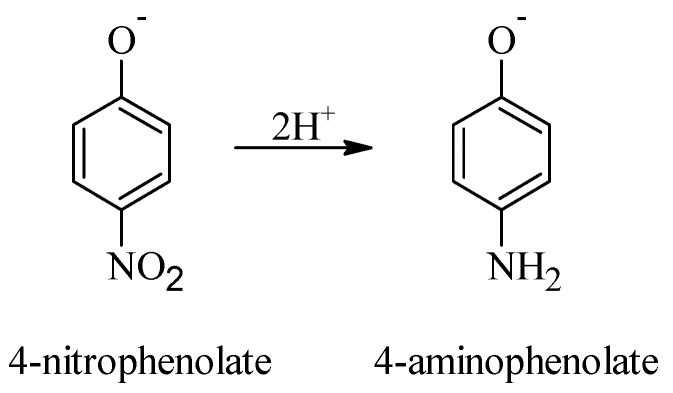
Mechanism for reduction of 4-nitrophenolate.

These results are in agreement with those obtained in the UV–vis spectrophotometry experiments (see the supplementary material), where the presence of a shoulder was observed in the UV region at around 265 nm simultaneously with the reduction of the characteristic maxima absorbance of 4-NP which recorded values close to zero. The formation of the secondary product is attributed to incomplete mineralization of 4-NP; and the nature of this secondary product can be inferred based on the results of additional ion chromatography experiments. Table 1 shows the results of IC analysis for nitrate quantification. Here, one can observe the presence of nitrate in the sample collected at the beginning of the experiments and a decrease in its concentration over the degradation time; this implies the occurrence of a photochemical reaction in 4-nitrophenolate with the release of nitrite, which is subsequently oxidized to nitrate in the presence of dissolved oxygen in solution. Furthermore, the results obtained point to the occurrence of the conversion of 4-nitrophenolate to 4-benzoquinone [34] in a portion of the total amount of the initial analyte (see Scheme 3). 4-benzoquinone is another byproduct that is possibly formed in the process, as indicated by the characteristic absorbance signal at around 270 nm [35,36] (see Figure S3 in the supplementary material). Here, it is reasonably likely that a parallel reaction occurs on the counter electrode, where 4-nitrophenolate is electrochemically reduced to 4-aminophenolate (see Scheme 4). 

The mineralization of 4-NP was evaluated based on the results obtained from TOC analysis. Aliquots of the working solution were collected and subjected to total organic carbon (TOC) quantification. The initial value of TOC, which was 18.38 mg∙L^−1^, diminished to 8.99 mg∙L^−1^; this represented 49.8% reduction of the total amount of 4-NP present in the sample prior to the degradation process. Indirectly, BiVO_4_/CuO was found to exhibit photoelectrocatalytic activity for the oxidation of 50.2% of initial 4-NP by •OH generation via water oxidation and electrolysis. 

Considering the presence of 4-NP as recalcitrant pollutant [36,37,38] in the environment, the photoanode proposed here has presented good performance and satisfactory reliability. The findings of the study reinforce the relevance and suitability of the technique employed here as a promising mechanism for the removal of 4-NP molecules in water treatment plants. 

## 4. Conclusions

The present work reported the development of a novel hybrid material BiVO_4_/CuO, which was successfully synthesized and characterized. The hybrid material presented homogeneous distribution between the polycrystalline structures of each constituent, as well as a suitable absorption edge value (1.85 eV) for its application as photoelectrocatalyzer in 4-NP degradation. The formation of *p-n* heterojunctions led to a decrease in the recombination rate of holes and electrons generated in the semiconductor. With regard to the application of the photoanode, the holes from the valence band of CuO were found to oxidize the water molecules adsorbed on the surface of the semiconductor, leading to the production of •OH at same time that water electrolysis occurred in the photoanode experimental data obtained from UV–vis, TOC, and IC analyses confirmed the hypothesis regarding the formation of secondary products due to photochemical reaction (i.e., 4-benzoquinone). Additionally, based on the results, it is possible to infer that 4-nitrophenolate undergoes electrochemical reduction reaction on the counter electrode, leading to the formation of 4-aminophenolate. The decrease of 50.2% in TOC, observed after the degradation process, shows that the proposed BiVO_4_/CuO is a promising material suitable for application as photoelectrocatalyzer in water treatment systems.

## Figures and Tables

**Table 1:** Data of nitrate concentration as function of degradation time, obtained through ion chromatography

Degradation Time/min	Nitrate/mg∙L^−1^
0	0.39
15	0.38
30	0.36
45	0.36
60	0.38
90	0.38
120	0.40
180	0.41
240	0.41
300	0.45
360	0.44
420	0.48

## Supplementary Materials

The following are available online at https://www.mdpi.com/1996-1944/13/6/1322/s1, Figure S1: Electrochemical cell and Led driver kit used to performer photoelectrochemical characterization of BiVO_4_/CuO. (a) Electrochemical cell with quartz window, (b) Counter electrode (platinum foil), (c) Working electrode (FTO/BiVO_4_/CuO), (d) Reference electrode (Ag/AgCl, KCl 3 mol∙L^−1^), (e) Potentiostat, (f) Led driver, and (g) White led (λ > 410 nm, 44 W); Figure S2: Tauc plots obtained from DRS data of (a) BiVO_4_, (b) BiVO_4_/CuO, and (c) CuO; Figure S3: Absorbance spectra of 4-nitrophenol obtained during their degradation.
