# Supplementary material for: Using BiVO4/CuO-Based Photoelectrocatalyzer for 4-Nitrophenol Degradation
Source: Materials (Basel). 2020 Mar 14;13(6):1322. doi: 10.3390/ma13061322 (PMC7143035; doi:10.3390/ma13061322)
Supplement: Supplementary file 1 [file materials-13-01322-s001.pdf]

materials

MDPI

Supplementary Material

Using $BiVO_4$/CuO-based photoelectrocatalyzer for 4-nitrophenol degradation

Thiago Martimiano do Prado, Fernando Lindo Silva, Guilherme Grosseli, Pedro Sergio Fadini, Orlando Fatibello-Filho and Fernando Cruz de Moraes *

Department of Chemistry, Federal University of São Carlos, São Carlos-SP 13565-905, Brazil; thipra@gmail.com(T.P.); fernandolindosilva@gmail.com(F.S.); guilhermegrosseli@gmail.com(G.G.); psfadini@gmail.com(P.F.); bello@ufscar.br (O.F.)

* Correspondence: fcmoraes@ufscar.br

Received: 21 February 2020; Accepted: 12 March 2020; Published: date

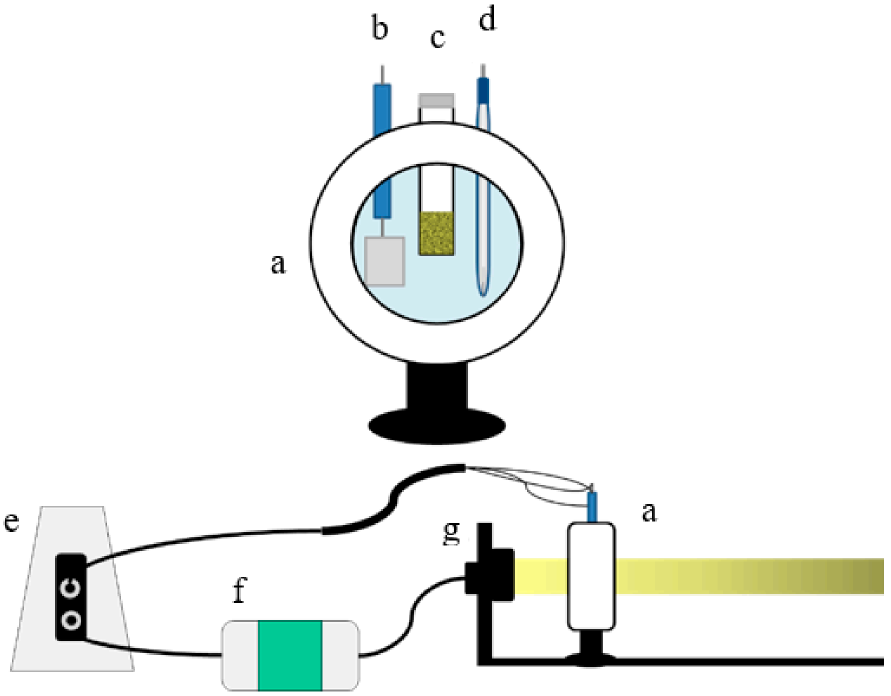

Figure S1. Electrochemical cell and Led driver kit used to performer photoelectrochemical characterization of $BiVO_4$/CuO. (**a**) Electrochemical cell with quartz window; (**b**) Counter electrode (platinum foil); (**c**) Working electrode (FTO/$BiVO_4$/CuO); (**d**) Reference electrode (Ag/AgCl, KCl 3 mol·L^{-1}); (**e**) Potenciostat; (**f**) Led driver and (**g**) White led ($\lambda > 410$ nm, 44 W).

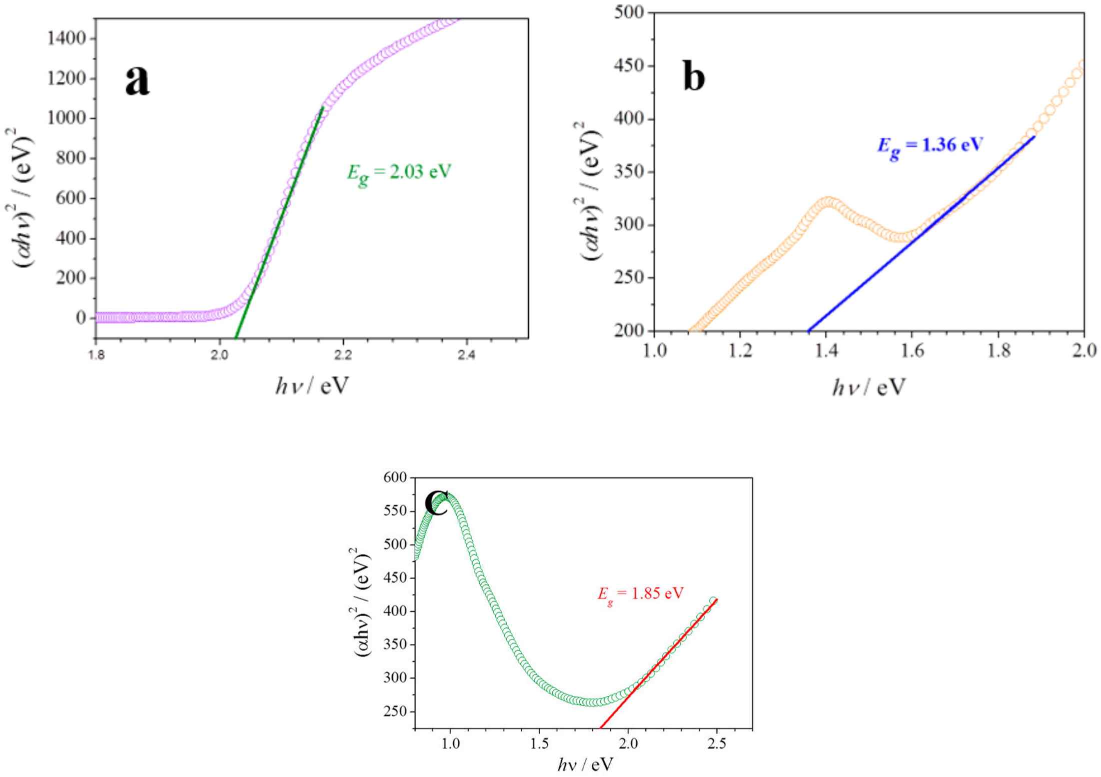

Figure S2. Tauc plots obtained from DRS data of (**a**) $BiVO_4$ (**b**) $BiVO_4$/CuO and (**c**) CuO.

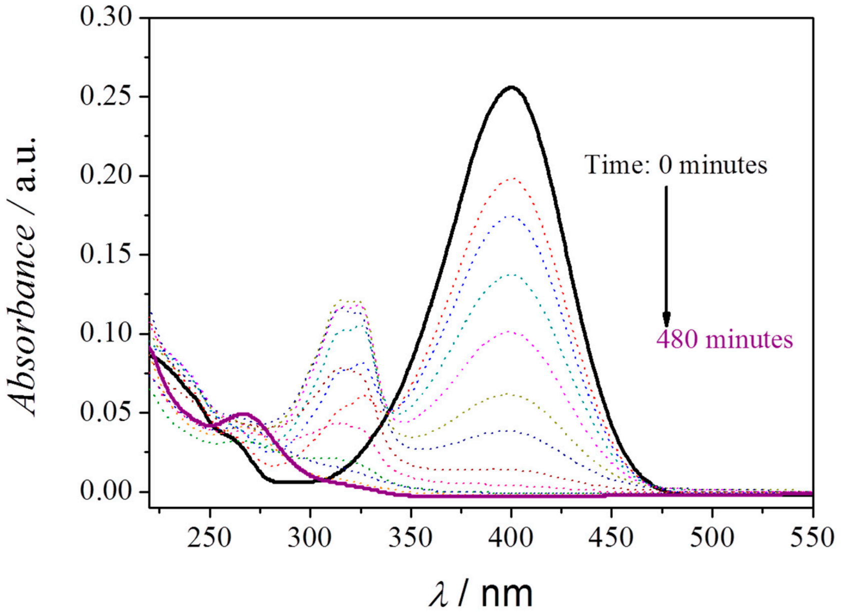

Figure S3. Absorbance spectra of 4-nitrophenol obtained during their degradation.

© 2020 by the authors. Licensee MDPI, Basel, Switzerland. This article is an open access article distributed under the terms and conditions of the Creative Commons Attribution (CC BY) license (http://creativecommons.org/licenses/by/4.0/).
